# Ameliorating effects of *Clerodendrum viscosum* leaves on lead‐induced hepatotoxicity

**DOI:** 10.1002/fsn3.4285

**Published:** 2024-06-18

**Authors:** Ehsanul Kabir, Jahidul Islam, Tasnim Tabassum Shila, Sharmin Akter Beauty, Junayed Sadi, Md. Royhan Gofur, Farhadul Islam, Shakhawoat Hossain, Farjana Nikkon, Khaled Hossain, Zahangir Alam Saud

**Affiliations:** ^1^ Department of Biochemistry and Molecular Biology Rajshahi University Rajshahi Bangladesh; ^2^ Department of Veterinary and Animal Sciences Rajshahi University Rajshahi Bangladesh

**Keywords:** antioxidant enzymes, hepatotoxicity, IL‐6, Nrf2, Pb exposure

## Abstract

Lead (Pb), a common toxicant is ubiquitously present in the environment. Chronic Pb exposure affects almost every organ system of human body including liver. *Clerodendrum viscosum* is a medicinal plant and its leaves are known to have hepatoprotective, anti‐inflammatory, and anti‐hyperglycemic activities. However, the protective effect of *C. viscosum* leaves against Pb‐induced hepatotoxicity is yet to be studied. Therefore, this study was designed to assess the protective effect of the aqueous extract of *C. viscosum* leaf (Cle) against Pb‐induced hepatotoxicity in experimental mice. Pb‐acetate was given to Pb and Pb + Cle groups interperitoneally, and Cle was supplemented to Cle and Pb + Cle groups by oral gavage. Serum biomarkers of liver function—butyrylcholinesterase (BChE), alkaline phosphatase (ALP), aspartate aminotransferase (AST), and alanine transaminase (ALT), antioxidant enzyme activities in hepatic tissue—superoxide dismutase (SOD), reduced glutathione reductase (rGR) and catalase (CAT), levels of transcription factor—nuclear factor erythroid 2‐related factor 2 (Nrf2), and inflammatory marker—interleukin‐6 (IL‐6) were analyzed. Additionally, histological analyses of hepatic tissues of all groups of experimental mice were performed. Pb‐treatment significantly increased ALP, AST, and ALT activities and decreased BChE activity compared to control mice. The antioxidant enzyme (SOD, rGR, and CAT) activities and expression of Nrf2 level were significantly (*p* < .05) decreased, while IL‐6 level was significantly (*p* < .05) increased in the hepatic tissue homogenates of Pb‐treated mice compared to the control group. Furthermore, histological examination revealed the disruption of hepatic tissue integrity in Pb‐treated mice. Notably, supplementation of Cle provided significant protection against the changes in the activities of liver function biomarkers and antioxidant enzymes, levels of Nrf2 and IL‐6, and disruption of hepatic tissue by Pb. Taken together the present study suggests that Cle ameliorates the hepatic toxicity caused by Pb.

## Graphical abstract

Lead (Pb), a common toxicant is ubiquitously present in environment. Chronic Pb exposure affects almost every organ system of human body including liver. Supplementation of Clerodendrum viscosum leaves provided significant protection against the changes in the activities of liver function biomarkers and antioxidant enzymes, levels of Nrf2 and IL‐6, and disruption of hepatic tissue by lead (Pb).

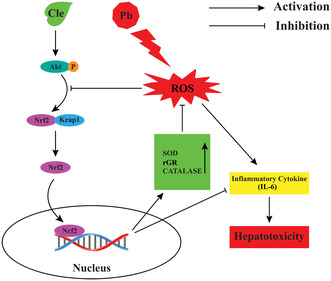

## 1 INTRODUCTION

Groundwater is the main source of drinking and irrigation water in Bangladesh, and most of the rural people use it as drinking water (Dey et al., 2017; Liong et al., 2000; Saha et al., 2018). There are a number of metals such as copper and manganese, which are essential to humans, however, their excessive presence can be toxic. Whereas some metals are highly toxic at very low concentrations and are harmful to human health, for example, arsenic, mercury, cadmium, and lead (Saha et al., 2017). Lead (Pb) is a common trace element that causes health problems in Bangladesh and several reports noted the presence of excessive amounts of Pb in the groundwater of different parts of Bangladesh (Banna et al., 2022; Bhuiyan et al., 2010; Mostafa et al., 2017). Pb exposure is known to be toxic to most organs such as the liver, kidneys, heart, brain, testes, and hematopoietic system (Abdel‐Moneim, 2012; Assi et al., 2016; Ilesanmi et al., 2022; Mujaibel & Kilarkaje, 2015). Pb exposure usually occurs in several ways – by consuming food and water contaminated with Pb, by breathing contaminated air, through dust, and by burning fuels and fossils. The liver is a major storage organ that has been shown to accumulate Pb in mice (Aktar et al., 2017; Flora et al., 2012). Pb exposure alters enzyme activity as well as molecules related to xenobiotic metabolism, cholesterol metabolism, and liver hyperplasia in Pb‐exposed experimental animals. Previous studies showed that Pb disrupts enzyme activation, prevents structural protein synthesis and absorption of trace elements, and reduces quantity and activity of antioxidants (Ercal et al., 2001; Patrick, 2006). In addition, Pb exposure has been reported to cause cellular and tissue damage in the liver, and there is a correlation between hepatotoxicity and blood Pb levels in laboratory animals (Nigra et al., 2016). Furthermore, Pb exposure impairs the structural integrity of the liver and ultimately causes hepatic disorders in experimental animals (Offor et al., 2017; Valko et al., 2005). Also, exposure to Pb facilitates the initiation of ROS‐induced oxidative stress with parallel depletion of antioxidant enzymes (Bokara et al., 2008; Gurer & Ercal, 2000). Chelation therapy is currently used to treat Pb‐poisoning with drugs, although chelators have side effects when treated with high doses for a long time (Offor et al., 2017). Using plant materials or consuming vegetables instead of chelators would be a good alternative to reduce Pb‐induced health effects.

Globally, plants have been used for various purposes, especially as human food and medications for various diseases and ailments from the very beginning of human history. Herbs have crucial roles in maintaining human well‐being and general health by eliminating nutritional deficiencies and restoring normal body functions. Also, herbal medicine is one of the most effective treatment options in the mainstream health‐care system. *Clerodendrum viscosum* is a plant, widely distributed in tropical regions, especially in India and Bangladesh. It is well known for its medicinal usage and extracts of different parts of the plant have been used in Ayurveda and Unani medicine to treat various diseases and ailments (Rahmatullah et al., 2011; Singh et al., 2018). Previous studies reported that *Clerodendrum viscosum* contains several secondary metabolites such as triterpenoids, flavonoid glycosides, flavonoids, iridoid glycosides, steroids, phenylpropanoids and neo‐clerodane diterpenes (Ghosh et al., 2015; Islam et al., 2023). Accordingly, *C. viscosum* phytochemicals showed anti‐inflammatory, antioxidant, anti‐infective, anti‐cancer, antibacterial, hyperglycemic, neuroprotective, and hepatoprotective potentials (Ahmed et al., 2007; Gouthamchandra et al., 2010; Haque et al., 2000; Rahman & Rumzhum, 2011; Rahmatullah et al., 2012; Sannigrahi et al., 2009). In addition, fresh *C. viscosum* leaf juice is used as a traditional cough syrup, anthelmintic, emetic, mild laxative, and decongestant in Bangladesh and India (Nandi & Mawkhlieng Lyndem, 2016). Also, *C. viscosum* leaf juice exhibits potent antioxidant and antiproliferative activities due to the presence of numerous bioactive compounds such as saponins, tannic acid, quercetin, ellagic acid, gallic acid, and reserpine (Shendge et al., 2017). Furthermore, several studies have shown that plant materials having antioxidant potential effectively diminish Pb‐mediated toxicity in experimental animals (Hsu & Guo, 2002; Jiao et al., 2011). For example, a recent study from our group revealed that *C. viscosum* leaf's aqueous extract prevents Pb‐mediated neurotoxicity in experimental animals (Islam et al., 2023). Thus, the presence of various bioactive compounds in *C. viscosum* leaves have been reported, however, the efficacy of aqueous extract of *C. viscosum* leaf in Pb‐mediated hepatic toxicity has not been extensively explored. Thus, herein we aimed to assess the protecting effects of aqueous extract of *C. viscosum* leaf against Pb‐induced hepatic toxicity in mice.

## 2 MATERIALS AND METHODS

### 2.1 Preparation of aqueous extract and qualitative phytochemical screening of *Clerodendrum viscosum* leaves

*Clerodendrum viscosum* young leaves were identified (Sample No. AA 107) by a taxonomist (Department of Botany, Rajshahi University) followed by collection from the Rajshahi University campus area, Bangladesh. In addition, botanical names have been verified in the “Plant List” by accessing http://www.theplantlist.org database. Aqueous extract of the leaves was prepared following the procedure described in the previous article (Islam et al., 2023). Briefly, after removing the petiole, the leaves were crushed to make a paste with double distilled water using a mortar and pestle. Leaf debris was removed using a muslin cloth, and the soluble fraction was collected and diluted to 1.5 mg/mL. *Clerodendrum viscosum* leaf extract (Cle) was kept in an airtight container (4°C) until used in the present study. Aqueous extract of leaves of *C. viscosum* has been used for the presence of various phytochemicals as previously described (Islam et al., 2023).

### 2.2 Animal maintenance and experimental design

Male Swiss Albino mice (25 ± 2 gm) were collected from icddr,b (International Centre for Diarrheal Disease Research, Bangladesh) and maintained in controlled conditions (25 ± 2°C) under natural light and dark cycles as described previously (Islam et al., 2022). After 7 days of acclimatization, 32 mice were distributed into four groups: (i) control, (ii) Pb‐treated, (iii) Cle‐treated, and (iv) Pb + Cle‐treated groups. Pb‐acetate was administered to Pb and Pb + Cle groups mice at the dose of 10 mg/kg‐bw by intraperitoneal (i.p.) injection for 1 month (30 days), while physiological saline was administered to control and Cle group as vehicle. During the experiment, an oral gavage tube was used to provide *C. viscosum* leaf (50 mg/kg‐bw) in Cle and Pb + Cle groups mice. At the same time, distilled water was given to the control and Pb‐treated mice as vehicle. Doses of Pb and Cle were selected as described in previously published articles (Aktar et al., 2017; Islam et al., 2023). All experimental mice received icddr,b formulated rodent food pellet and drinking water as ad libitum during the entire experimental period. Experiments for animal studies were accomplished according to the guidelines permitted by the recognized ethics board (No: 255(14)/320(1)/IAMEBBC/IBSc), Rajshahi University, Bangladesh.

### 2.3 Collection of blood specimen and preparation of hepatic tissue homogenate from experimental mice

Blood was collected from thoracic artery of the experimental animals followed by diethyl ether anesthesia. Serum was then collected, followed by clotting by centrifugation at 4°C, 1610 *g* for 15 min, and stored at −80°C. For liver tissue collection, the whole liver was excised, washed, marked, and measured the weight with electronic balance. Then, phosphate buffer (0.1 M; pH 7) and Triton X100 (0.5%) solution (Sigma Aldrich, USA) were used to prepare tissue homogenization. Finally, the supernatant of the tissue homogenate was collected after centrifuging at 4°C at 2516 *g* for 15 min and used to examine the biochemical parameters (Biswas et al., 2019; Reza et al., 2018).

### 2.4 Biochemical assay of serum liver enzymes

Kits for activity assay of BChE (RANDOX, UK), AST, ALT, and ALP (Human Diagnostics, Germany) were used to analyze the activity of the respective enzymes in serum by analyzer (Humalyzer‐3000, Germany) following their instructions.

### 2.5 Biochemical assay of antioxidant enzymes, Nrf2 and IL‐6 from liver tissues

The activities of SOD, rGR, and CAT, and total protein in homogenate of liver tissue were measured using the protocol described in Islam et al. (2022) and Bonaventura et al. (1972). ELISA kits (Elabscience, USA) were used to measure Nrf2 and IL‐6 proteins in liver homogenate following manufacturer's guidelines.

### 2.6 Histopathological changes in the liver tissues

Livers of experimental mice (two from each group) were washed carefully using saline water. Then, the tissues were placed in formalin (10% v/v solution) for organ fixation. The samples were then embedded in paraffin followed by dehydrated with ethanol. Five micrometers of liver sections were obtained from the paraffin‐embedded samples using a microtome. Then, a standard pathological staining method was used on the tissue sections and finally, a light microscope was used to examine the changes in the tissue slices (Biswas et al., 2019).

### 2.7 Statistical analysis

Results are presented as mean ± standard error of the mean (SEM). Welch's test followed by one‐way ANOVA was used to determine the statistical significance among the experimental groups, where *p* < .05 was considered to be statistically significant. GraphPad Prism version 7.05 was used to analyze the data and graph illustration.

## 3 RESULTS

### 3.1 Existence of various phytochemicals in Cle

Screening of phytochemical analysis showed that the *C. viscosum* leaf's aqueous extract contains saponins, phenolic compounds, alkaloids, flavonoids, and tannins. Phytochemical screening data were incorporated from our recently published article (Islam et al., 2023).

### 3.2 Serum BChE activity in Cle‐supplemented Pb‐exposed mice

**FIGURE 1:**
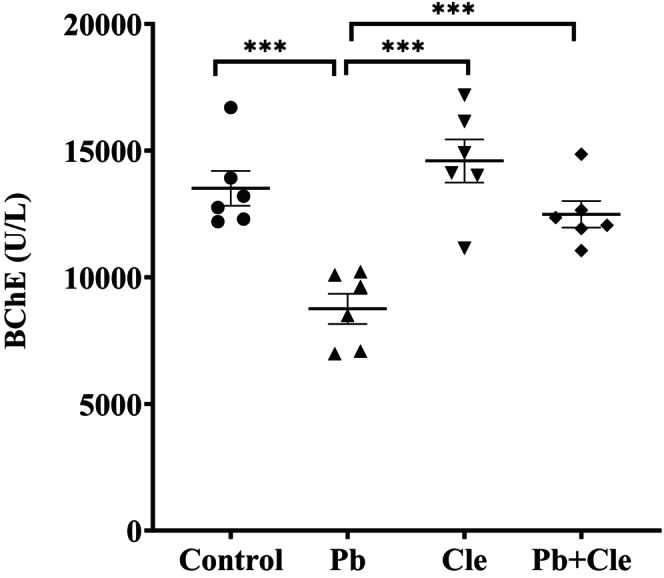
Cle ameliorates the decrease in serum BChE activity in Pb‐exposed mice. Control, lead (Pb), Cle, and Pb + Cle mice were expressed as mean ± SEM, where *n* = 6 for each group of mice. Significantly different means were performed by Welch's *t*‐test (***p < .001) and ordinary one‐way ANOVA (*p* < .0001).

Butyrylcholinesterase (BChE) is produced in the liver and its serum levels have been shown to be prognostic markers of liver disease. The activity of BChE in serum of experimental mice groups are presented in Figure 1. Activity of BChE in the serum of mice groups (control, Pb‐treated, Cle, and Pb + Cle) were 13,514 ± 687.80, 8754 ± 598, 14,594 ± 849, and 12,485 ± 523.80 U/L, respectively. BChE activity was reduced in serum of mice exposed to Pb in comparison to that of control animals (*p* < .001). While Cle supplementation enhances BChE activity in Pb + Cle‐treated mice in comparison to Pb‐exposed animals (*p* < .001).

### 3.3 Cle prevented the alterations of ALP, AST, and ALT levels in the blood of Pb‐treated mice

Chronic exposure to toxic substances leads to alter liver enzyme activity in serum. The effect of Cle on ALP, AST, and ALT levels in the serum of the experimental mice was presented in Table 1. ALP levels in serum were 116.3 ± 4.08, 137.4 ± 4.601, 90.7 ± 3.849, and 104.8 ± 4.442 U/L in control, Pb‐treated, Cle, and Pb + Cle groups, respectively. Similarly, ALT for control, Pb‐treated, Cle, and Pb + Cle groups were 53.72 ± 5.756, 84.10 ± 4.729, 52.72 ± 2.52, and 61.45 ± 2.209 U/L, respectively, while AST activity was 62.92 ± 4.453, 96.78 ± 4.367, 59.40 ± 5.523 and 68.38 ± 2.034 U/L, respectively in above mentioned groups. A remarkable increase of serum ALP, ALT, and AST levels was noted in Pb‐exposed mice compared to control mice (*p* < .01). Importantly, a significant restoration of these enzymes' activities was noted in Pb‐exposed mice receiving Cle supplementation compared to only the Pb‐treated mice group (*p* < .01).

**TABLE 1:** Serum ALP, AST and ALT activities of the experimental mice.

Liver enzymes activity(U/L)	Experimental groups				*p*‐value
	Control	Pb	Cle	Pb + Cle	
ALP	116.3 ± 4.08	137.4 ± 4.601^a^	90.7 ± 3.847^b^	104.8 ± 4.442^c^	^a^ *p* = .0065, ^b^ *p* < .0001, ^c^ *p* = .0005
AST	62.92 ± 4.453	96.78 ± 4.367^a^	59.4 ± 5.523^b^	68.38 ± 2.034^c^	^a^ *p* = .0003, ^b^ *p* = .0004, ^c^ *p =* .0006
ALT	53.72 ± 5.756	84.1 ± 4.729^a^	52.72 ± 2.52^b^	61.45 ± 2.209^c^	^a^ *p* = .0024, ^b^ *p* = .0005, ^c^ *p* = .0033

*Note*: Data are expressed as mean ± SEM, *n* = 6 for each group of mice. ^a^Significantly different from control, and ^b,c^Significantly different from lead (Pb) mice group. Group comparisons were performed by Welch's *t*‐test and ordinary one‐way ANOVA (*p* < .0001).

### 3.4 Antioxidant enzyme activity in liver tissue of experimental mice

Table 2 showed the antioxidant enzymes (SOD, rGR, and CAT) activities in the liver of four experimental groups. In liver tissue, the activity of SOD in mice of four groups (control, Pb‐treated, Cle, and Pb + Cle) was 4.84 ± 0.11, 3.46 ± 0.22, 5.11 ± 0.17, and 4.71 ± 0.05 U/mg, respectively. SOD activity was decreased in the liver homogenate of the Pb‐exposed group in comparison to that of the control group (*p* < .001) and Cle supplementation significantly elevated the SOD activity in liver tissue of Pb + Cle animals than that of Pb‐treated mice (*p* < .01). Activity of rGR was 0.204 ± 0.006, 0.147 ± 0.006, 0.208 ± 0.005, and 0.180 ± 0.004 umol/mg, respectively in liver tissue of all four groups. Result showed that Pb exposure remarkably reduced rGR activity than that of control group (*p* < .0001). However, significantly restored rGR activity was observed in the liver homogenate of Pb‐exposed mice receiving Cle supplementation (*p* < .01). Also, activity of CAT was found 41.03 ± 1.97 μmol/mg in control, 31.66 ± 1.29 umol/mg in Pb‐treated, 42.35 ± 1.86 umol/mg in Cle‐treated and 39.79 ± 1.22 μmol/mg in Pb + Cle‐treated mice group. Pb exposure reduced the CAT activity whereas Cle supplementation restored CAT activity.

**TABLE 2:** Liver tissue GR, SOD, and catalase activities of the experimental mice.

Enzyme activity	Experimental groups				*p*‐value
	Control	Pb	Cle	Pb + Cle	
rGR (μmol/mg)	0.204 ± 0.006	0.147 ± 0.006^a^	0.208 ± 0.005^b^	0.180 ± 0.004^c^	^a^ *p* < .0001, ^b^ *p* < .0001, ^c^ *p* = .0019
SOD (U/mg)	4.84 ± 0.11	3.46 ± 0.22	5.11 ± 0.17^b^	4.71 ± 0.05^c^	^a^ *p* = .0007, ^b^ *p* = .002, ^c^ *p* = .0019
Catalase (μmol/mg)	41.03 ± 1.97	31.66 ± 1.29 ^a^	42.35 ± 1.86^b^	39.79 ± 1.22^c^	^a^ *p* = .0035, ^b^ *p* = .0011, ^c^ *p* = .001

*Note*: Data are expressed as mean ± SEM, *n* = 6 for each group of mice. ^a^Significantly different from control, and ^b,c^Significantly different from lead (Pb) mice group. Group comparisons were performed by Welch's *t*‐test and ordinary one‐way ANOVA (*p* < .0001).

### 3.5 Restoring effects of Cle on Nrf2 and IL‐6 in the liver tissue of Pb‐induced mice

**FIGURE 2:**
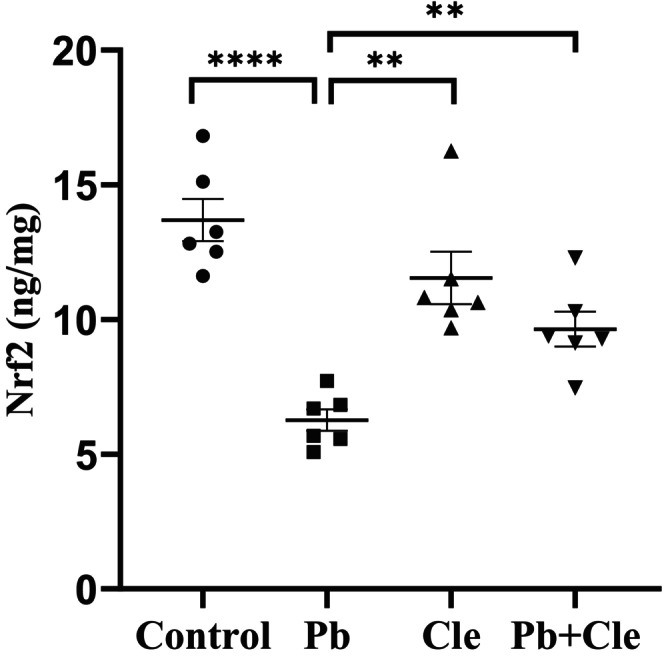
Cle supplementation increased Nrf2 levels in Pb‐exposed mice. Control (C), lead (Pb), Cle, and Pb + Cle mice were expressed as mean ± SEM, where *n* = 6 for each group of mice. Significantly different among means were conducted by Welch's *t*‐test (**p < .01, ****p < .0001) and ordinary one‐way ANOVA (*p* < .0001).

**FIGURE 3:**
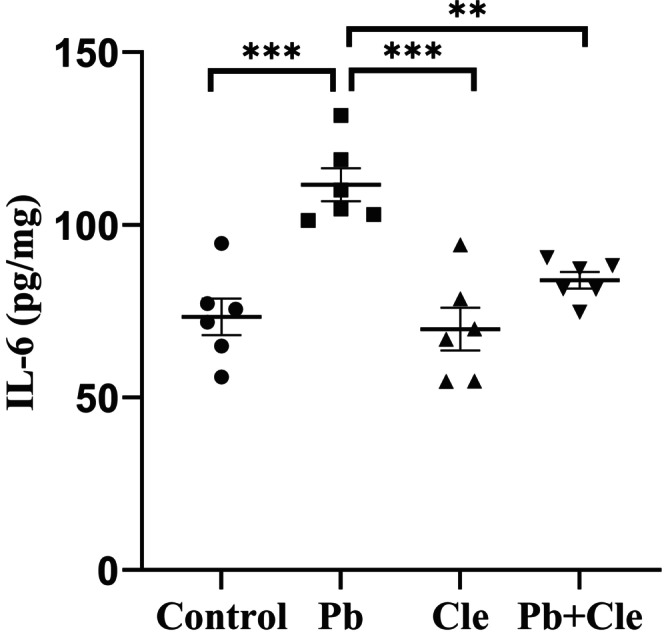
Cle supplementation reduced IL‐6 levels in liver tissue of Pb‐exposed mice. Control (C), lead (Pb), Cle, and Pb + Cle mice were expressed as mean ± SEM, where *n* = 6 for each group of mice. Significantly different means were conducted by Welch's t‐test (***p < .001, **p < .01) and ordinary one‐way ANOVA (*p* < .0001).

We measured Nrf2 levels in the liver tissue of the experimental groups to estimate the ameliorative effect of Cle against Pb‐induced disturbance of the antioxidant system. Nrf2 levels in hepatic tissue of the experimental animals are presented in Figure 2. Nrf2 levels in the liver homogenate were 13.16 ± 0.78, 6.26 ± 0.40, 11.55 ± 0.97, and 9.64 ± 0.65 ng/mg in control, Pb‐treated, Cle, and Pb + Cle groups, respectively. A significant decreased level of Nrf2 was found in liver tissue of Pb‐treated mice group compared to control mice (*p* < .0001). Cle supplement to mice group pre‐exposed to Pb reestablished levels of Nrf2 significantly (*p* < .01). In addition, we analyzed the levels of inflammatory factor IL‐6 in the liver tissue homogenate of the experimental mice groups. IL‐6 levels of mice were in control (73.32 ± 5.318), Pb‐treated (111.60 ± 4.786), Cle (69.78 ± 6.159) and Pb + Cle (83.92 ± 2.38) pg/mg (Figure 3). Thus, Pb exposure increased IL‐6 levels in liver tissue significantly (*p* < .001), which indicates Pb‐induce severe inflammation. Interestingly Cle supplementation significantly decreased the IL‐6 level in Pb‐exposed mice (*p* < .01) followed by upregulation of Nrf2 level.

### 3.6 Cle attenuated Pb‐treated histopathological alterations in liver

**FIGURE 4:**
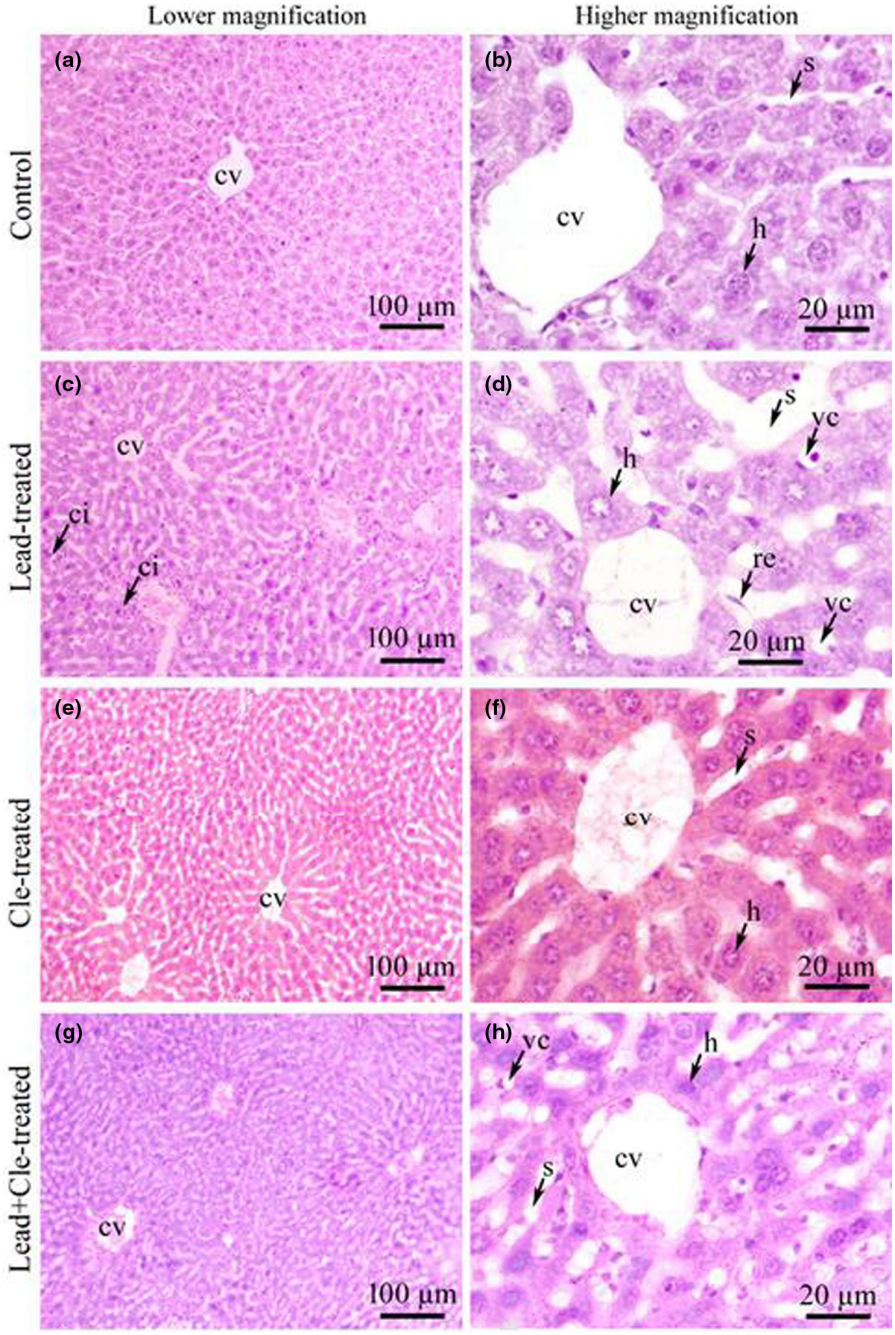
Histomicrographs of hepatic tissue of experimental mice (H&E stain). Liver tissues of control mice showed central veins (cv) with normal intact hepatocytes (h) surrounding it, no vacuolations (a, b). Liver sections of lead‐treated mice (Pb) showed extensive cellular infiltration (ci) around the portal areas with some hepatocyte necrosis, vacuolation or vacuolar degeneration (vc), endothelial rupture (re), wide sinusoidal space (s) with hepatocytes (c, d). *Clerodendrum viscosum* leaf‐treated mice (Cle) showed central veins (cv) with normal intact hepatocytes (h) surrounding it, no vacuolations; same as control mice (e, f). Liver sections from Pb + Cle‐treated mice showed central veins (cv) with normal intact hepatocytes (h) with minor vacuolations (vc) (g, h). Scale bar 100 μm (a, c, e, g; lower magnification) and 20 μm (b, d, f, h; higher magnification).

Histopathological examination showed the structure of regular hepatocytes with the sinusoidal areas in liver tissue in control mice (Figure 4a,b). No histopathological changes were noted in Cle‐supplemented animal groups (Figure 4e,f). However, massive cellular infiltration throughout the portal area with necrosis of some hepatocytes, vacuolar degeneration in hepatocytes, wide sinusoidal spaces with endothelial rupture, and hepatocytic enlargement and degeneration were detected in liver sections of Pb‐exposed mice (Figure 4c,d). Notable improved pathological features were noted in mice pre‐exposed to Pb receiving Cle supplementation (Figure 4g,h).

## 4 DISCUSSION

Liver is the most important and largest human organ for drug metabolism and detoxification. Exposure to Pb has been associated with significant health issues, particularly the failure of many organs due to the alteration of oxidative stress. Most importantly, liver is the most vulnerable organ, where Pb accumulates and damages the liver tissues and its functionality. Therefore, Pb exposure in experimental animals and humans can result in a variety of physiological, biochemical, and behavioral problems. Using a mouse model, we have examined here the preventive properties of *C. viscosum* leaf (Cle) against Pb‐induced liver injury. The current study's findings demonstrated that Cle supplementation attenuated the adverse effects of Pb on the hepatic system in Pb‐exposed mice by restoring the cellular‐antioxidant defense system. Plants materials are rich in flavonoids, which significantly reduce metal‐induced hepatic toxicities of experimental animals (Abdel‐Moneim et al., 2011; Gong et al., 2014). Accordingly, in a recent study, we have shown that *C. viscosum* leaf contains pharmacological bioactive compounds such as flavonoids and saponin, thereby showing a significant neuroprotective potential in Pb‐exposed mice (Islam et al., 2023).

Butyrylcholinesterase (BChE) is a serum marker to indicate the state of liver inflammation and Pb exposure is associated with decreased BChE levels in serum (Ogunkeye & Roluga, 2016). It is also reported that various clinical conditions such as chronic and acute hepatic dysfunction, inflammation, and infection decrease serum BChE levels (Santarpia et al., 2013). This reduced level of BChE in serum indicates many clinical conditions of the liver disorder, including infection, inflammation, injury, and both acute and chronic liver damage. (Aktar et al., 2017; Banna et al., 2022; Zivkovic et al., 2015). Similarly, in the present study, a lower BChE level was measured in the serum of Pb‐treated mice than that of control group of mice, and BChE levels in the serum of Pb‐exposed mice were restored upon Cle supplementation. In addition, enzyme activity levels (ALP, AST, and ALT) are potential markers for liver integrity and functions. Pb exposure is associated with elevation of these enzymes in serum due to acute hepatotoxicity or mild hepatocellular injury (Giannini et al., 2005). Previously we reported that serum level of ALP, AST, and ALT were elevated in mice exposed to Pb, which implied that hepatic function was impaired in Pb‐exposed mice in comparison to the mice of control group (Aktar et al., 2017; Banna et al., 2022). Current study results, that is, Pb‐induced deterioration of hepatic enzymes are consistent with the previous reports. Elevated levels of liver enzymes in the serum of Pb‐treated mice are due to the impairment of the structural integrity of the liver (Offor et al., 2017). As noted in this study, the presence of histopathological abnormalities, that is, degeneration, necrosis, and extensive infiltration in the liver of Pb‐exposed mice were observed whereas Cle supplementation could potentially replenish these abnormalities in the liver of Pb exposure mice, thereby restored the integrity of liver tissues and functionality. It is assumed that the phytochemicals of the supplemental Cle could play active roles in protecting the liver structure and function against Pb‐induced toxicity.

In liver diseases, the most important pathogenic phenomena are inflammation and oxidative stress. Oxidative stress is a result of an imbalance between the generation of free radicals and biological systems' capacity to detoxify reactive intermediates (free radicals) (Flora, 2011; Xu et al., 2019). It is stated that Pb exposure prohibits liver antioxidant enzyme activities in laboratory animals (Abdel‐Moneim et al., 2011). According to the previous studies, the liver tissues of Pb‐treated animals demonstrated significantly reduced activity of SOD, CAT, and rGR than those of control animals. Increased production of reactive oxygen species (ROS) as a result of damage to the enzymes involved in mitochondrial respiration could be the mechanism underlying Pb‐induced oxidative stress. Also, previous studies reported that Pb exposure influences excessive ROS production, which ultimately depletes antioxidant reserved in cells (Abdel‐Moneim et al., 2011; Ercal et al., 2001). Moreover, chronic Pb exposure is associated with higher lipid peroxidation, thereby producing increased amount of ROS and disrupting the antioxidant defense system across multiple organs in experimental animals (Kasperczyk et al., 2015). It is interesting to note that antioxidant‐rich medicinal plants have been used to stop oxidative stress‐related diseases in humans. (Sharifi‐Rad et al., 2020). Thus, the pharmacological agents and plant extracts possessing metal binding and antioxidant properties can be used to neutralize Pb‐induced oxidative stress. The antioxidant activity noted in *C. viscosum* leaf could attributed to maintaining the endogenous antioxidant system, thereby reducing the oxidative stress and alleviating Pb‐induced pathological changes in the liver of experimental mice. In earlier studies, we and other groups have shown that *C. viscosum* leaves are rich in antioxidant phytochemicals such as phenolic compounds, which can alleviate the metal‐induced neurotoxicity in mice (Islam et al., 2023; Shendge et al., 2017). Therefore, it is presumed that Cle supplementation has demonstrated notable hepatoprotective features by boosting the antioxidant system in Pb‐exposed mice due to the presence of potent antioxidant and bioactive compounds in it.

The transcription factor Nrf2 is crucial for cellular resistance to exogenous toxins and oxidative stress (He et al., 2020). Antioxidant and anti‐inflammatory therapies are useful in the treatment of liver diseases where Nrf2 acts as an important factor in cell defense mediated by antioxidant and anti‐inflammatory responses. The development of chronic inflammatory diseases is associated with dysregulation of Nrf2 activity (Xu et al., 2019). Previous reports demonstrated that Nrf2 was downregulated in brain, kidney, and liver of chronically Pb‐exposed laboratory animals (Hu et al., 2020; Islam et al., 2023; Liu et al., 2017; Zhang et al., 2021). Consistent with the previous findings, we also found a reduction of Nrf2 level in liver tissue of mice exposed to Pb indicating antioxidant system dysfunction in the liver compared to control mice group. Interestingly, mice pre‐exposed to Pb receiving Cle‐supplementation significantly returned Nrf2 levels in liver tissue. Therefore, antioxidant compounds in Cle could nullify Pb‐mediated ROS generation in mice liver by activation of Nrf2‐mediated antioxidant systems. Earlier, Dai et al. (2021) reported that saponin extract from *Panax japonicas* elevates Akt phosphorylation and activates Nrf2 to attenuate CCL4‐induced liver fibrosis in mice. Zhou et al. (2014) also reported that saponins in the leaves of *Panax notoginseng* exert antioxidant effects through Nrf2 activation to prevent brain cell injury from H_2_O_2_‐induced oxidative stress. Similarly, in our previous study, we demonstrated the presence of a higher amount of saponin in the aqueous extract of *C. viscosum* leaf which can influence the restoration of Nrf2 level in the brain of the mice exposed to Pb (Islam et al., 2023).

**FIGURE 5:**
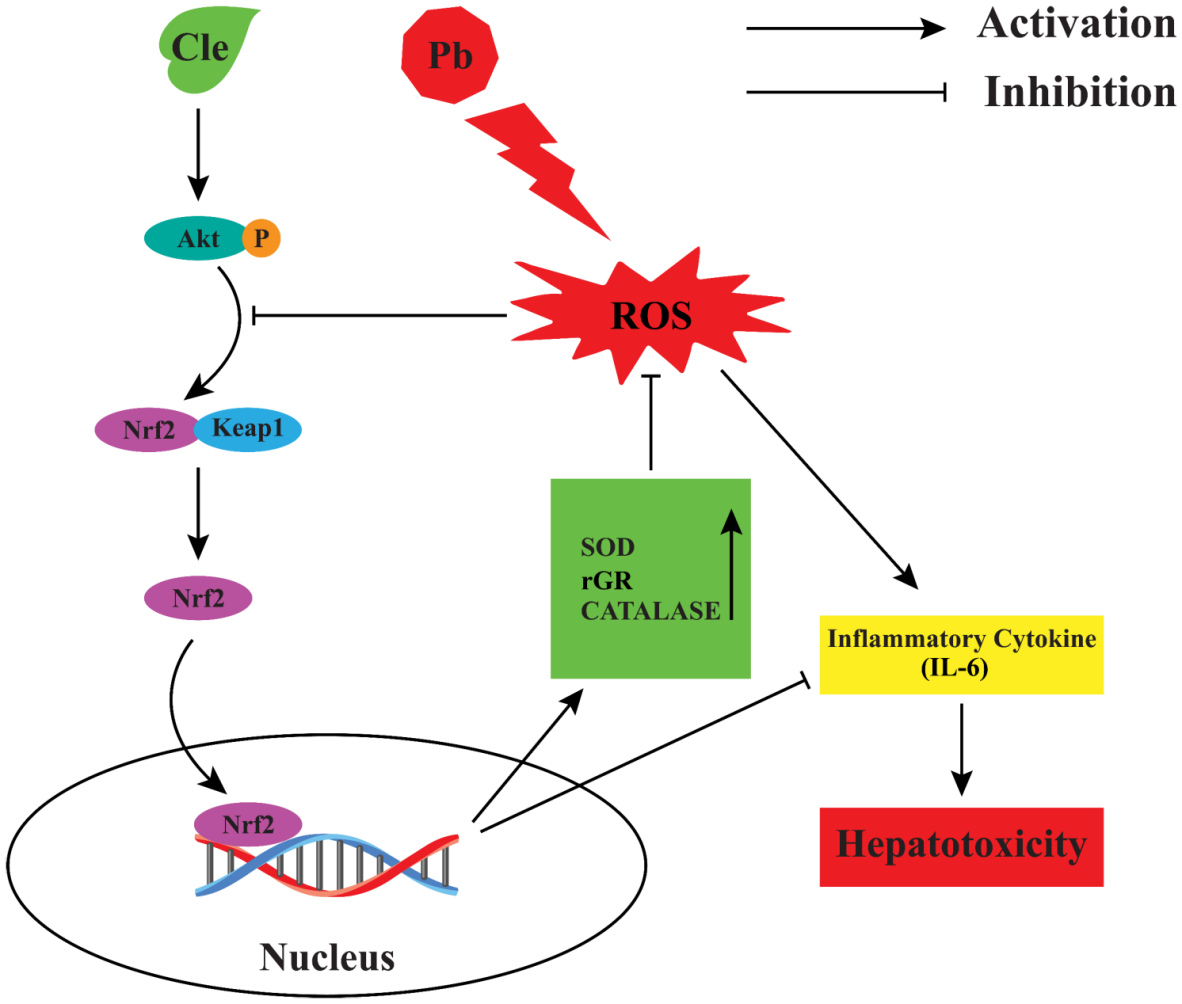
Possible mechanisms of the protective effect of Cle on Pb‐induced hepatotoxicity in mice. Chronic Pb exposure generates excessive oxidative stress and enhanced inflammation through interruption Nrf2 antioxidative system in liver. Supplementation of Cle decreased the IL‐6 level by upregulation of Nrf2 in the liver.

Inflammation and oxidative stress are frequently linked to liver damage, and it has been suggested that oxidative stress increases or activates the inflammatory response by upregulating genes related to inflammation (Shanmugam et al., 2016; Tan et al., 2018). IL‐6 is a well‐known pro‐inflammatory cytokine and has been extensively studied. According to previous reports, Pb exposure causes tissue damage and inflammation by inhibiting anti‐inflammatory mechanisms in experimental animals. It also increases the production of pro‐inflammatory cytokines, such as IL‐6 (Chang et al., 2013; Dietert et al., 2004; Hsiao et al., 2011). In addition, previous studies revealed that Nrf2 activation inhibits pro‐inflammatory cytokine IL‐6 gene expression in human macrophages (Kobayashi et al., 2016; Zinovkin & Grebenchikov, 2020). In this study, higher IL‐6 levels and reduced Nrf2 levels were noted in liver tissue of mice exposed to Pb when compared to the mice of control group, which implied the hepatic impairment induced by Pb exposure. Interestingly, Cle supplementation significantly reduced IL‐6 level and enhanced Nrf2 level in mice exposed to Pb, indicating improved hepatic functions. In addition, tissue damages induced by Pb exposure were alleviated by Cle supplementation as indicated by less histopathological changes in comparison to the mice exposed to Pb. Therefore, the active phytochemicals such as rutin, gallic acid, and saponin, in leaves of *C. viscosum* could induce the production of Nrf2 through Akt phosphorylation in Pb‐pre‐exposed mice receiving Cle supplementation, resulting in nullifying IL‐6‐mediated liver damages. Thus, the biologically active substances in leaves of *C. viscosum* might promote the activation of antioxidant system through Nrf2 upregulation, which attenuates inflammation, resulting in the activation of Nrf2‐HO‐IL‐6 pathway and finally preventing hepatotoxicity in Pb‐exposed mice (Figure 5). Nevertheless, further studies are needed to elucidate the exact molecular mechanism of *C. viscosum* leaf extract‐mediated protection against Pb‐induced hepatotoxicity in experimental animals. Moreover, the quantitative damage in histological slide was not analyzed in this study, and in a good sense, we have no reservations in stating that this may be a specific limitation of this study.

## 5 CONCLUSION

*Clerodendrum viscosum* leaf provides significant protection in mice against Pb‐induced hepatic dysfunctions. Supplementation of Cle boosted up Nrf2 level in Pb‐treated mice, thus, restoring the antioxidant system. Therefore, this animal study suggested that aqueous extract of *C. viscosum* leaf may be used to remediate Pb‐induced toxicity in humans.

## AUTHOR CONTRIBUTIONS

**Ehsanul Kabir:** Formal analysis (equal); investigation (equal); writing – original draft (equal). **Jahidul Islam:** Formal analysis (equal); investigation (equal); writing – original draft (equal). **Tasnim Tabassum Shila:** Data curation (equal); investigation (equal). **Sharmin Akter Beauty:** Data curation (equal); investigation (equal). **Junayed Sadi:** Data curation (equal); investigation (equal). **Md. Royhan Gofur:** Investigation (equal); methodology (equal). **Farhadul Islam:** Writing – review and editing (equal). **Shakhawoat Hossain:** Investigation (equal); methodology (equal). **Farjana Nikkon:** Funding acquisition (equal); validation (equal). **Khaled Hossain:** Writing – review and editing (equal). **Zahangir Alam Saud:** Conceptualization (equal); funding acquisition (equal); methodology (equal); project administration (equal); supervision (equal); writing – original draft (equal).

## CONFLICT OF INTEREST STATEMENT

None.

## ETHICAL APPROVAL

The Institution Ethics, Biosafety, and Biosecurity Committee approved (No. 255(14)/320(1)/IAMEBBC/IBSc) the protocols and methods used in this study (Rajshahi University, Bangladesh).

## Data Availability

The data of the study is available from the corresponding author on request.
